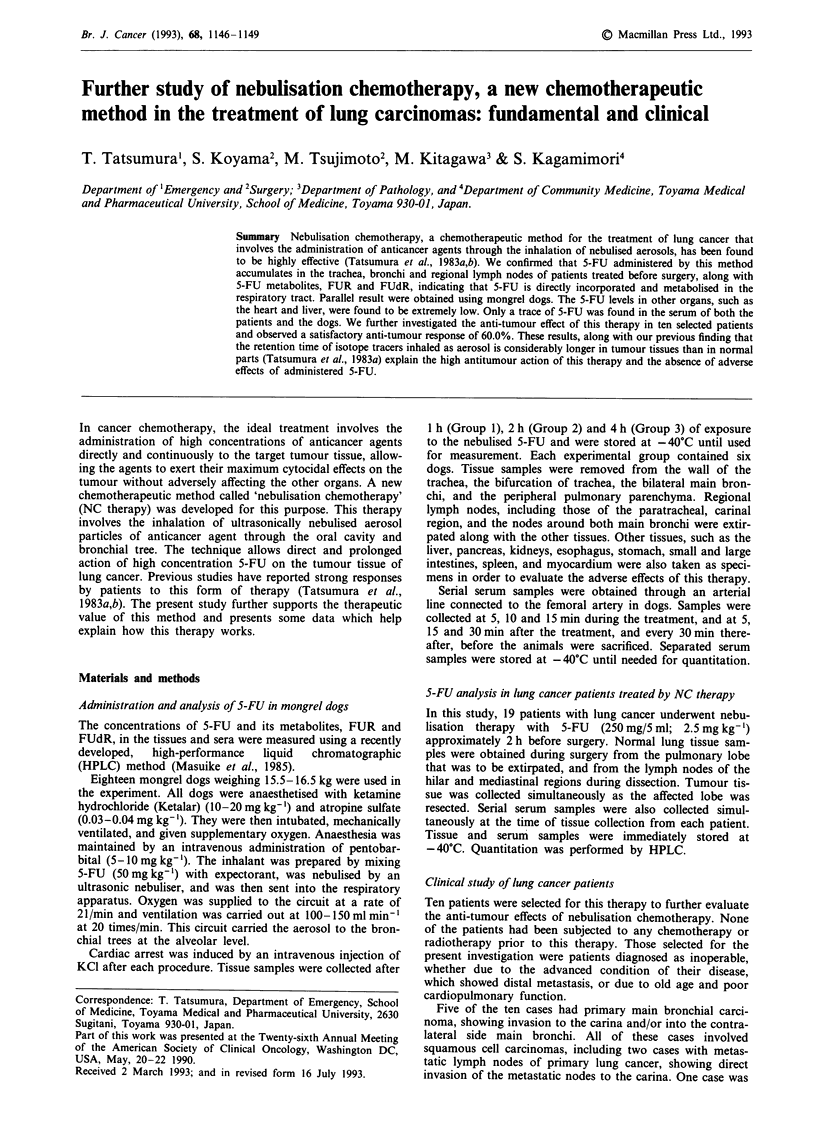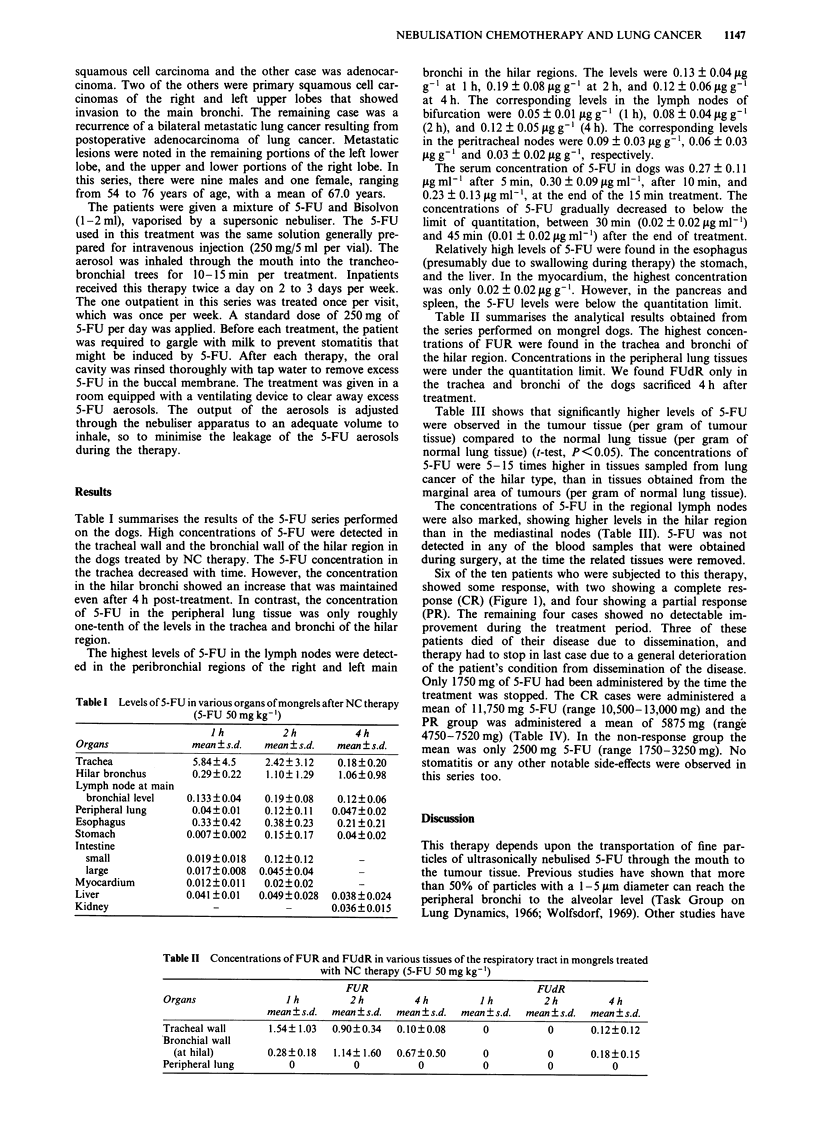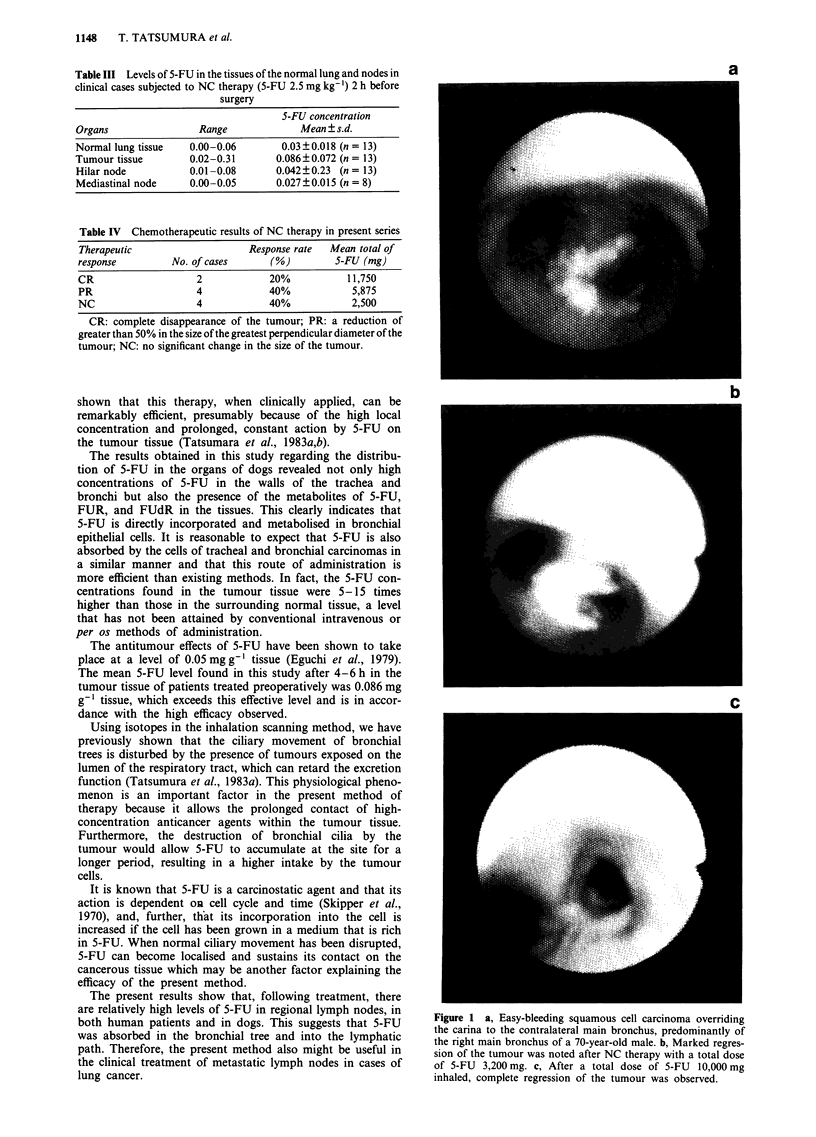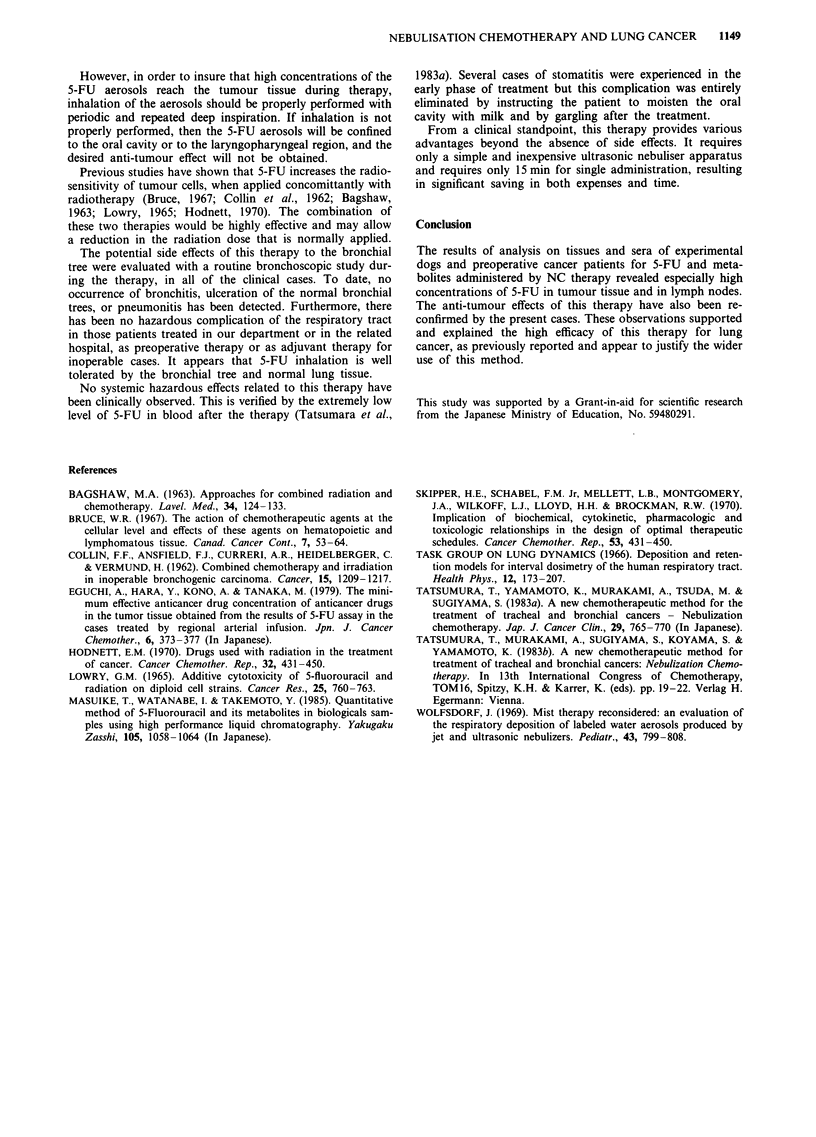# Further study of nebulisation chemotherapy, a new chemotherapeutic method in the treatment of lung carcinomas: fundamental and clinical.

**DOI:** 10.1038/bjc.1993.495

**Published:** 1993-12

**Authors:** T. Tatsumura, S. Koyama, M. Tsujimoto, M. Kitagawa, S. Kagamimori

**Affiliations:** Department of Emergency, Toyama Medical and Pharmaceutical University, School of Medicine, Japan.

## Abstract

**Images:**


Br. J. Cancer (1993), 68, 1146–1149 © Macmillan Press Ltd., 1993

### Further study of nebulisation chemotherapy, a new chemotherapeutic method in the treatment of lung carcinomas: fundamental and clinical

T. Tatsumura[1], S. Koyama[2], M. Tsujimoto[2], M. Kitagawa[3] & S. Kagamimori[4]

*Department of [1]Emergency and [2]Surgery; [3]Department of Pathology, and [4]Department of Community Medicine, Toyama Medical and Pharmaceutical University, School of Medicine, Toyama 930-01, Japan.*

**Summary** Nebulisation chemotherapy, a chemotherapeutic method for the treatment of lung cancer that involves the administration of anticancer agents through the inhalation of nebulised aerosols, has been found to be highly effective (Tatsumura *et al.*, 1983*a*,*b*). We confirmed that 5-FU administered by this method accumulates in the trachea, bronchi and regional lymph nodes of patients treated before surgery, along with 5-FU metabolites, FUR and FUdR, indicating that 5-FU is directly incorporated and metabolised in the respiratory tract. Parallel result were obtained using mongrel dogs. The 5-FU levels in other organs, such as the heart and liver, were found to be extremely low. Only a trace of 5-FU was found in the serum of both the patients and the dogs. We further investigated the anti-tumour effect of this therapy in ten selected patients and observed a satisfactory anti-tumour response of 60.0%. These results, along with our previous finding that the retention time of isotope tracers inhaled as aerosol is considerably longer in tumour tissues than in normal parts (Tatsumura *et al.*, 1983*a*) explain the high antitumour action of this therapy and the absence of adverse effects of administered 5-FU.

In cancer chemotherapy, the ideal treatment involves the administration of high concentrations of anticancer agents directly and continuously to the target tumour tissue, allowing the agents to exert their maximum cytocidal effects on the tumour without adversely affecting the other organs. A new chemotherapeutic method called 'nebulisation chemotherapy' (NC therapy) was developed for this purpose. This therapy involves the inhalation of ultrasonically nebulised aerosol particles of anticancer agent through the oral cavity and bronchial tree. The technique allows direct and prolonged action of high concentration 5-FU on the tumour tissue of lung cancer. Previous studies have reported strong responses by patients to this form of therapy (Tatsumura *et al.*, 1983*a*,*b*). The present study further supports the therapeutic value of this method and presents some data which help explain how this therapy works.

### Materials and methods

### *Administration and analysis of 5-FU in mongrel dogs*

The concentrations of 5-FU and its metabolites, FUR and FUdR, in the tissues and sera were measured using a recently developed, high-performance liquid chromatographic (HPLC) method (Masuike *et al.*, 1985).

Eighteen mongrel dogs weighing 15.5–16.5 kg were used in the experiment. All dogs were anaesthetised with ketamine hydrochloride (Ketalar) (10–20 mg $kg^{-1}$) and atropine sulfate (0.03–0.04 mg $kg^{-1}$). They were then intubated, mechanically ventilated, and given supplementary oxygen. Anaesthesia was maintained by an intravenous administration of pentobarbital (5–10 mg $kg^{-1}$). The inhalant was prepared by mixing 5-FU (50 mg $kg^{-1}$) with expectorant, was nebulised by an ultrasonic nebuliser, and was then sent into the respiratory apparatus. Oxygen was supplied to the circuit at a rate of 21/min and ventilation was carried out at 100–150 ml $min^{-1}$ at 20 times/min. This circuit carried the aerosol to the bronchial trees at the alveolar level.

Cardiac arrest was induced by an intravenous injection of KCl after each procedure. Tissue samples were collected after 1 h (Group 1), 2 h (Group 2) and 4 h (Group 3) of exposure to the nebulised 5-FU and were stored at −40°C until used for measurement. Each experimental group contained six dogs. Tissue samples were removed from the wall of the trachea, the bifurcation of trachea, the bilateral main bronchi, and the peripheral pulmonary parenchyma. Regional lymph nodes, including those of the paratracheal, carinal region, and the nodes around both main bronchi were extirpated along with the other tissues. Other tissues, such as the liver, pancreas, kidneys, esophagus, stomach, small and large intestines, spleen, and myocardium were also taken as specimens in order to evaluate the adverse effects of this therapy.

Serial serum samples were obtained through an arterial line connected to the femoral artery in dogs. Samples were collected at 5, 10 and 15 min during the treatment, and at 5, 15 and 30 min after the treatment, and every 30 min thereafter, before the animals were sacrificed. Separated serum samples were stored at −40°C until needed for quantitation.

### *5-FU analysis in lung cancer patients treated by NC therapy*

In this study, 19 patients with lung cancer underwent nebulisation therapy with 5-FU (250 mg/5 ml; 2.5 mg $kg^{-1}$) approximately 2 h before surgery. Normal lung tissue samples were obtained during surgery from the pulmonary lobe that was to be extirpated, and from the lymph nodes of the hilar and mediastinal regions during dissection. Tumour tissue was collected simultaneously as the affected lobe was resected. Serial serum samples were also collected simultaneously at the time of tissue collection from each patient. Tissue and serum samples were immediately stored at −40°C. Quantitation was performed by HPLC.

### *Clinical study of lung cancer patients*

Ten patients were selected for this therapy to further evaluate the anti-tumour effects of nebulisation chemotherapy. None of the patients had been subjected to any chemotherapy or radiotherapy prior to this therapy. Those selected for the present investigation were patients diagnosed as inoperable, whether due to the advanced condition of their disease, which showed distal metastasis, or due to old age and poor cardiopulmonary function.

Five of the ten cases had primary main bronchial carcinoma, showing invasion to the carina and/or into the contralateral side main bronchi. All of these cases involved squamous cell carcinomas, including two cases with metastatic lymph nodes of primary lung cancer, showing direct invasion of the metastatic nodes to the carina. One case was

Correspondence: T. Tatsumura, Department of Emergency, School of Medicine, Toyama Medical and Pharmaceutical University, 2630 Sugitani, Toyama 930-01, Japan.
Part of this work was presented at the Twenty-sixth Annual Meeting of the American Society of Clinical Oncology, Washington DC, USA, May, 20–22 1990.
Received 2 March 1993; and in revised form 16 July 1993.

squamous cell carcinoma and the other case was adenocarcinoma. Two of the others were primary squamous cell carcinomas of the right and left upper lobes that showed invasion to the main bronchi. The remaining case was a recurrence of a bilateral metastatic lung cancer resulting from postoperative adenocarcinoma of lung cancer. Metastatic lesions were noted in the remaining portions of the left lower lobe, and the upper and lower portions of the right lobe. In this series, there were nine males and one female, ranging from 54 to 76 years of age, with a mean of 67.0 years.

The patients were given a mixture of 5-FU and Bisolvon (1–2 ml), vaporised by a supersonic nebuliser. The 5-FU used in this treatment was the same solution generally prepared for intravenous injection (250 mg/5 ml per vial). The aerosol was inhaled through the mouth into the trancheobronchial trees for 10–15 min per treatment. Inpatients received this therapy twice a day on 2 to 3 days per week. The one outpatient in this series was treated once per visit, which was once per week. A standard dose of 250 mg of 5-FU per day was applied. Before each treatment, the patient was required to gargle with milk to prevent stomatitis that might be induced by 5-FU. After each therapy, the oral cavity was rinsed thoroughly with tap water to remove excess 5-FU in the buccal membrane. The treatment was given in a room equipped with a ventilating device to clear away excess 5-FU aerosols. The output of the aerosols is adjusted through the nebuliser apparatus to an adequate volume to inhale, so to minimise the leakage of the 5-FU aerosols during the therapy.

### Results

Table I summarises the results of the 5-FU series performed on the dogs. High concentrations of 5-FU were detected in the tracheal wall and the bronchial wall of the hilar region in the dogs treated by NC therapy. The 5-FU concentration in the trachea decreased with time. However, the concentration in the hilar bronchi showed an increase that was maintained even after 4 h post-treatment. In contrast, the concentration of 5-FU in the peripheral lung tissue was only roughly one-tenth of the levels in the trachea and bronchi of the hilar region.

The highest levels of 5-FU in the lymph nodes were detected in the peribronchial regions of the right and left main bronchi in the hilar regions. The levels were 0.13 ± 0.04 μg $g^{-1}$ at 1 h, 0.19 ± 0.08 μg $g^{-1}$ at 2 h, and 0.12 ± 0.06 μg $g^{-1}$ at 4 h. The corresponding levels in the lymph nodes of bifurcation were 0.05 ± 0.01 μg $g^{-1}$ (1 h), 0.08 ± 0.04 μg $g^{-1}$ (2 h), and 0.12 ± 0.05 μg $g^{-1}$ (4 h). The corresponding levels in the peritracheal nodes were 0.09 ± 0.03 μg $g^{-1}$, 0.06 ± 0.03 μg $g^{-1}$ and 0.03 ± 0.02 μg $g^{-1}$, respectively.

The serum concentration of 5-FU in dogs was 0.27 ± 0.11 μg $ml^{-1}$ after 5 min, 0.30 ± 0.09 μg $ml^{-1}$, after 10 min, and 0.23 ± 0.13 μg $ml^{-1}$, at the end of the 15 min treatment. The concentrations of 5-FU gradually decreased to below the limit of quantitation, between 30 min (0.02 ± 0.02 μg $ml^{-1}$) and 45 min (0.01 ± 0.02 μg $ml^{-1}$) after the end of treatment.

Relatively high levels of 5-FU were found in the esophagus (presumably due to swallowing during therapy) the stomach, and the liver. In the myocardium, the highest concentration was only 0.02 ± 0.02 μg $g^{-1}$. However, in the pancreas and spleen, the 5-FU levels were below the quantitation limit.

Table II summarises the analytical results obtained from the series performed on mongrel dogs. The highest concentrations of FUR were found in the trachea and bronchi of the hilar region. Concentrations in the peripheral lung tissues were under the quantitation limit. We found FUdR only in the trachea and bronchi of the dogs sacrificed 4 h after treatment.

Table III shows that significantly higher levels of 5-FU were observed in the tumour tissue (per gram of tumour tissue) compared to the normal lung tissue (per gram of normal lung tissue) ($t$-test, $P<0.05$). The concentrations of 5-FU were 5–15 times higher in tissues sampled from lung cancer of the hilar type, than in tissues obtained from the marginal area of tumours (per gram of normal lung tissue).

The concentrations of 5-FU in the regional lymph nodes were also marked, showing higher levels in the hilar region than in the mediastinal nodes (Table III). 5-FU was not detected in any of the blood samples that were obtained during surgery, at the time the related tissues were removed.

Six of the ten patients who were subjected to this therapy, showed some response, with two showing a complete response (CR) (Figure 1), and four showing a partial response (PR). The remaining four cases showed no detectable improvement during the treatment period. Three of these patients died of their disease due to dissemination, and therapy had to stop in last case due to a general deterioration of the patient's condition from dissemination of the disease. Only 1750 mg of 5-FU had been administered by the time the treatment was stopped. The CR cases were administered a mean of 11,750 mg 5-FU (range 10,500–13,000 mg) and the PR group was administered a mean of 5875 mg (range 4750–7520 mg) (Table IV). In the non-response group the mean was only 2500 mg 5-FU (range 1750–3250 mg). No stomatitis or any other notable side-effects were observed in this series too.

### Discussion

This therapy depends upon the transportation of fine particles of ultrasonically nebulised 5-FU through the mouth to the tumour tissue. Previous studies have shown that more than 50% of particles with a 1–5 μm diameter can reach the peripheral bronchi to the alveolar level (Task Group on Lung Dynamics, 1966; Wolfsdorf, 1969). Other studies have

**Table I** Levels of 5-FU in various organs of mongrels after NC therapy (5-FU 50 mg $kg^{-1}$)

| *Organs* | *1 h mean ± s.d.* | *2 h mean ± s.d.* | *4 h mean ± s.d.* |
|---|---|---|---|
| Trachea | 5.84±4.5 | 2.42±3.12 | 0.18±0.20 |
| Hilar bronchus | 0.29±0.22 | 1.10±1.29 | 1.06±0.98 |
| Lymph node at main bronchial level | 0.133±0.04 | 0.19±0.08 | 0.12±0.06 |
| Peripheral lung | 0.04±0.01 | 0.12±0.11 | 0.047±0.02 |
| Esophagus | 0.33±0.42 | 0.38±0.23 | 0.21±0.21 |
| Stomach | 0.007±0.002 | 0.15±0.17 | 0.04±0.02 |
| Intestine | | | |
| small | 0.019±0.018 | 0.12±0.12 | – |
| large | 0.017±0.008 | 0.045±0.04 | – |
| Myocardium | 0.012±0.011 | 0.02±0.02 | – |
| Liver | 0.041±0.01 | 0.049±0.028 | 0.038±0.024 |
| Kidney | – | – | 0.036±0.015 |

**Table II** Concentrations of FUR and FUdR in various tissues of the respiratory tract in mongrels treated with NC therapy (5-FU 50 mg $kg^{-1}$)

| *Organs* | *FUR 1 h mean ± s.d.* | *FUR 2 h mean ± s.d.* | *FUR 4 h mean ± s.d.* | *FUdR 1 h mean ± s.d.* | *FUdR 2 h mean ± s.d.* | *FUdR 4 h mean ± s.d.* |
|---|---|---|---|---|---|---|
| Tracheal wall | 1.54±1.03 | 0.90±0.34 | 0.10±0.08 | 0 | 0 | 0.12±0.12 |
| Bronchial wall (at hilal) | 0.28±0.18 | 1.14±1.60 | 0.67±0.50 | 0 | 0 | 0.18±0.15 |
| Peripheral lung | 0 | 0 | 0 | 0 | 0 | 0 |

**Table III** Levels of 5-FU in the tissues of the normal lung and nodes in clinical cases subjected to NC therapy (5-FU 2.5 mg $kg^{-1}$) 2 h before surgery

| *Organs* | *Range* | *5-FU concentration Mean ± s.d.* |
|---|---|---|
| Normal lung tissue | 0.00–0.06 | 0.03 ± 0.018 (*n* = 13) |
| Tumour tissue | 0.02–0.31 | 0.086 ± 0.072 (*n* = 13) |
| Hilar node | 0.01–0.08 | 0.042 ± 0.23 (*n* = 13) |
| Mediastinal node | 0.00–0.05 | 0.027 ± 0.015 (*n* = 8) |

**Table IV** Chemotherapeutic results of NC therapy in present series

| *Therapeutic response* | *No. of cases* | *Response rate (%)* | *Mean total of 5-FU (mg)* |
|---|---|---|---|
| CR | 2 | 20% | 11,750 |
| PR | 4 | 40% | 5,875 |
| NC | 4 | 40% | 2,500 |

CR: complete disappearance of the tumour; PR: a reduction of greater than 50% in the size of the greatest perpendicular diameter of the tumour; NC: no significant change in the size of the tumour.

shown that this therapy, when clinically applied, can be remarkably efficient, presumably because of the high local concentration and prolonged, constant action by 5-FU on the tumour tissue (Tatsumara *et al.*, 1983*a*,*b*).

The results obtained in this study regarding the distribution of 5-FU in the organs of dogs revealed not only high concentrations of 5-FU in the walls of the trachea and bronchi but also the presence of the metabolites of 5-FU, FUR, and FUdR in the tissues. This clearly indicates that 5-FU is directly incorporated and metabolised in bronchial epithelial cells. It is reasonable to expect that 5-FU is also absorbed by the cells of tracheal and bronchial carcinomas in a similar manner and that this route of administration is more efficient than existing methods. In fact, the 5-FU concentrations found in the tumour tissue were 5–15 times higher than those in the surrounding normal tissue, a level that has not been attained by conventional intravenous or *per os* methods of administration.

The antitumour effects of 5-FU have been shown to take place at a level of 0.05 mg $g^{-1}$ tissue (Eguchi *et al.*, 1979). The mean 5-FU level found in this study after 4–6 h in the tumour tissue of patients treated preoperatively was 0.086 mg $g^{-1}$ tissue, which exceeds this effective level and is in accordance with the high efficacy observed.

Using isotopes in the inhalation scanning method, we have previously shown that the ciliary movement of bronchial trees is disturbed by the presence of tumours exposed on the lumen of the respiratory tract, which can retard the excretion function (Tatsumura *et al.*, 1983*a*). This physiological phenomenon is an important factor in the present method of therapy because it allows the prolonged contact of high-concentration anticancer agents within the tumour tissue. Furthermore, the destruction of bronchial cilia by the tumour would allow 5-FU to accumulate at the site for a longer period, resulting in a higher intake by the tumour cells.

It is known that 5-FU is a carcinostatic agent and that its action is dependent on cell cycle and time (Skipper *et al.*, 1970), and, further, that its incorporation into the cell is increased if the cell has been grown in a medium that is rich in 5-FU. When normal ciliary movement has been disrupted, 5-FU can become localised and sustains its contact on the cancerous tissue which may be another factor explaining the efficacy of the present method.

The present results show that, following treatment, there are relatively high levels of 5-FU in regional lymph nodes, in both human patients and in dogs. This suggests that 5-FU was absorbed in the bronchial tree and into the lymphatic path. Therefore, the present method also might be useful in the clinical treatment of metastatic lymph nodes in cases of lung cancer.

**Figure 1** **a**, Easy-bleeding squamous cell carcinoma overriding the carina to the contralateral main bronchus, predominantly of the right main bronchus of a 70-year-old male. **b**, Marked regression of the tumour was noted after NC therapy with a total dose of 5-FU 3,200 mg. **c**, After a total dose of 5-FU 10,000 mg inhaled, complete regression of the tumour was observed.

However, in order to insure that high concentrations of the 5-FU aerosols reach the tumour tissue during therapy, inhalation of the aerosols should be properly performed with periodic and repeated deep inspiration. If inhalation is not properly performed, then the 5-FU aerosols will be confined to the oral cavity or to the laryngopharyngeal region, and the desired anti-tumour effect will not be obtained.

Previous studies have shown that 5-FU increases the radiosensitivity of tumour cells, when applied concomittantly with radiotherapy (Bruce, 1967; Collin *et al.*, 1962; Bagshaw, 1963; Lowry, 1965; Hodnett, 1970). The combination of these two therapies would be highly effective and may allow a reduction in the radiation dose that is normally applied.

The potential side effects of this therapy to the bronchial tree were evaluated with a routine bronchoscopic study during the therapy, in all of the clinical cases. To date, no occurrence of bronchitis, ulceration of the normal bronchial trees, or pneumonitis has been detected. Furthermore, there has been no hazardous complication of the respiratory tract in those patients treated in our department or in the related hospital, as preoperative therapy or as adjuvant therapy for inoperable cases. It appears that 5-FU inhalation is well tolerated by the bronchial tree and normal lung tissue.

No systemic hazardous effects related to this therapy have been clinically observed. This is verified by the extremely low level of 5-FU in blood after the therapy (Tatsumara *et al.*, 1983*a*). Several cases of stomatitis were experienced in the early phase of treatment but this complication was entirely eliminated by instructing the patient to moisten the oral cavity with milk and by gargling after the treatment.

From a clinical standpoint, this therapy provides various advantages beyond the absence of side effects. It requires only a simple and inexpensive ultrasonic nebuliser apparatus and requires only 15 min for single administration, resulting in significant saving in both expenses and time.

### Conclusion

The results of analysis on tissues and sera of experimental dogs and preoperative cancer patients for 5-FU and metabolites administered by NC therapy revealed especially high concentrations of 5-FU in tumour tissue and in lymph nodes. The anti-tumour effects of this therapy have also been reconfirmed by the present cases. These observations supported and explained the high efficacy of this therapy for lung cancer, as previously reported and appear to justify the wider use of this method.

This study was supported by a Grant-in-aid for scientific research from the Japanese Ministry of Education, No. 59480291.

### References

BAGSHAW, M.A. (1963). Approaches for combined radiation and chemotherapy. *Lavel. Med.*, **34**, 124–133.

BRUCE, W.R. (1967). The action of chemotherapeutic agents at the cellular level and effects of these agents on hematopoietic and lymphomatous tissue. *Canad. Cancer Cont.*, **7**, 53–64.

COLLIN, F.F., ANSFIELD, F.J., CURRERI, A.R., HEIDELBERGER, C. & VERMUND, H. (1962). Combined chemotherapy and irradiation in inoperable bronchogenic carcinoma. *Cancer*, **15**, 1209–1217.

EGUCHI, A., HARA, Y., KONO, A. & TANAKA, M. (1979). The minimum effective anticancer drug concentration of anticancer drugs in the tumor tissue obtained from the results of 5-FU assay in the cases treated by regional arterial infusion. *Jpn. J. Cancer Chemother.*, **6**, 373–377 (In Japanese).

HODNETT, E.M. (1970). Drugs used with radiation in the treatment of cancer. *Cancer Chemother. Rep.*, **32**, 431–450.

LOWRY, G.M. (1965). Additive cytotoxicity of 5-fluorouracil and radiation on diploid cell strains. *Cancer Res.*, **25**, 760–763.

MASUIKE, T., WATANABE, I. & TAKEMOTO, Y. (1985). Quantitative method of 5-Fluorouracil and its metabolites in biologicals samples using high performance liquid chromatography. *Yakugaku Zasshi*, **105**, 1058–1064 (In Japanese).

SKIPPER, H.E., SCHABEL, F.M. Jr, MELLETT, L.B., MONTGOMERY, J.A., WILKOFF, L.J., LLOYD, H.H. & BROCKMAN, R.W. (1970). Implication of biochemical, cytokinetic, pharmacologic and toxicologic relationships in the design of optimal therapeutic schedules. *Cancer Chemother. Rep.*, **53**, 431–450.

TASK GROUP ON LUNG DYNAMICS (1966). Deposition and retention models for interval dosimetry of the human respiratory tract. *Health Phys.*, **12**, 173–207.

TATSUMURA, T., YAMAMOTO, K., MURAKAMI, A., TSUDA, M. & SUGIYAMA, S. (1983*a*). A new chemotherapeutic method for the treatment of tracheal and bronchial cancers – Nebulization chemotherapy. *Jap. J. Cancer Clin.*, **29**, 765–770 (In Japanese).

TATSUMURA, T., MURAKAMI, A., SUGIYAMA, S., KOYAMA, S. & YAMAMOTO, K. (1983*b*). A new chemotherapeutic method for treatment of tracheal and bronchial cancers: *Nebulization Chemotherapy*. In 13th International Congress of Chemotherapy, TOM16, Spitzy, K.H. & Karrer, K. (eds). pp. 19–22. Verlag H. Egermann: Vienna.

WOLFSDORF, J. (1969). Mist therapy reconsidered: an evaluation of the respiratory deposition of labeled water aerosols produced by jet and ultrasonic nebulizers. *Pediatr.*, **43**, 799–808.